# Factors associated with parental resolution of a child's autism diagnosis: A systematic review

**DOI:** 10.3389/fpsyt.2022.1079371

**Published:** 2023-01-05

**Authors:** Vrinda V. Naicker, Simon M. Bury, Darren Hedley

**Affiliations:** Olga Tennison Autism Research Centre, School of Psychology and Public Health, La Trobe University, Melbourne, VIC, Australia

**Keywords:** acceptance, autism spectrum disorder, diagnosis, hope, parent-child relationship, parental wellbeing, resolution, systematic review

## Abstract

**Background:**

Receiving a child's autism diagnosis can be stressful; as such, parent resolution contributes to the wellbeing and development of healthy parent-child relationships. In other significant childhood diagnoses (e.g., cerebral palsy, diabetes), the degree to which parents adjust to (a) their child's diagnosis and (b) their changes in expectations concerning their child's development and capacity (referred to as resolution to diagnosis), has been associated with improved outcomes including facilitating parent-child relationships and improved parental wellbeing. Given potential benefits to parent and child, and the heterogenous nature of autism, examining the unique factors associated with resolution to diagnosis is important. In this systematic review we identified factors that support or inhibit parental resolution to their child receiving a diagnosis of autism.

**Methods:**

We completed a systematic review following PRISMA guidelines of peer-reviewed studies from 2017 to 2022, that investigated parental resolution or acceptance of an autism diagnosis. Papers including “acceptance” needed to encompass both accepting the diagnosis and the implications regarding the child's abilities. We searched six databases (Scopus, Web of Science, MEDLINE, PsycINFO, ProQuest), with additional papers located following review of reference lists.

**Results:**

Fourteen papers with 592 participants that investigated parental resolution or acceptance of an autism diagnosis, were included. We identified six common factors that facilitate or inhibit parental resolution and acceptance of an autism diagnosis including: symptom severity; religion, belief, and culture; knowledge and uncertainty; negative emotions (i.e., denial, shame, guilt); positive emotions; and support. Greater resolution was associated with improved “attunement and insightfulness” in the parent-child relationship.

**Limitation:**

The review was limited by the small number of studies meeting inclusion criteria. Second, the quality of included studies was mixed, with over half of the studies being qualitative and only one randomized control trial (RCT) identified.

**Conclusion:**

Parental resolution can have an impact on parent's perception of their child's capabilities and impact the parent-child relationship. We identified six categories that aid in inhibiting or promoting resolution to diagnosis. Despite taking a broad approach on the definition of resolution, the low number of studies identified in the review indicates a need for more research in this area.

**Systematic review registration:**

http://www.crd.york.ac.uk/PROSPERO/, PROSPERO [ID: CRD42022336283].

## 1. Introduction

Autism spectrum disorder (ASD; henceforth “autism”) is a neurodevelopmental condition, typically emerging in late infancy or early childhood (1). The Diagnostic and Statistical Manual of Mental Disorders (5th ed.; DSM−5) (2) diagnostic criteria for autism includes persistent differences in two domains: (a) social communication and interaction skills and (b) restricted repetitive patterns of behavior, interest, or activities. Given the potential for a diagnosis of autism to change one's expectations for their child, the process associated with a child's diagnosis can be particularly stressful for parents (3). Furthermore, there is potential for the nature of the parent-child relationship to change following the diagnosis, for example, by altering the parent's perception of their child's developmental and functional capacity, which may lead to additional impacts on child and parental wellbeing (4). However, this process of acceptance in which parents come to terms with and manage their new reality, referred to as “resolution of diagnosis”, has been associated with greater parental wellbeing (5, 6).

Resolution, a construct derived from attachment theory (7–10), involves understanding the child's diagnosis and the implications of the diagnosis on parents' internal representation of their child, from pre-diagnosis to post-diagnosis (10). Resolution requires parents to adjust their internal representations and expectations to the new reality of having a child who has been diagnosed with a neurodevelopmental disability (5) and adjusting their parental approach to match this reality. Internal representation refers to the expectations, feelings, and acknowledgment regarding the child's personality and behaviors (4). Important in this process is for parents to realistically understand and accept the challenges associated with a child's diagnosis to better facilitate the parent-child relationship and their support, and thus the wellbeing of both (11). Similar concepts of adjusting and acceptance of a diagnosis can be found in other theoretical approaches, such as Psychological Adjustment (12) and Expectation Management Theory of Acceptance (13), which also includes the process of accepting the implications of the child's diagnosis and has similar positive benefits on the parent child relationship (13, 14).

Benefits of resolution to parents of children with significant childhood diagnoses includes a greater capacity of parents to cope with stress, resulting in reduced psychological distress and depression, higher levels of marital satisfaction, and seeking and accessing social support (9, 14). For example, mothers of autistic children who are more emotionally resolved reported higher levels of cognitive and supportive engagement during play interactions with their child, provided greater verbal and non-verbal scaffolding skills (15), skills critical for the development of attention and play (16). However, the process of resolution is not a simple process, with unresolved parents often reporting denial or disbelief and searching for alternate explanations, for example, seeking a second opinion regarding their child's autism diagnosis (17). Factors including severity of diagnosis, parental nationality and age, knowledge, and negative emotions (e.g., guilt, grief, shame) have been found to negatively inhibit the process of resolution (3, 18, 19).

Several recent reviews have examined resolution or similar acceptance-based processes within the context of significant child diagnoses. Sher-Censor and Shahar-Lahav's (6) recent scoping review identified studies using the Reaction to Diagnosis Index (RDI) to examine parent's response to their child's diagnosis of a disability (inclusive of autism). They identified 13 studies involving parents of autistic children. Overall, authors found a lack of resolution for parents of children with a disability was common (44% of participants from 47 studies). They also found associations between lack of resolution and higher parenting stress, poor parental mental health, and insecure attachment with their child.

While not focused explicitly on resolution, Makino et al.'s (20) scoping review identified benefits associated with a similar process of acceptance in which acceptance included coming to terms with the new family circumstances and future needs of their child. Parents identified several benefits of receiving their child's autism diagnosis and raising an autistic child, included becoming more patient and less judgemental. Similarly, Brown et al.'s (21) narrative review of father's experiences revealed an ongoing period of adjustment to their child's autism diagnosis, leading to acceptance of the child and gratefulness for the knowledge and experiences gained. This period of adjustment was framed positively as leading to a stronger parental bond with their child. However, negative initial emotions or factors that might inhibit the process of acceptance (e.g., denial, confusion, shame, disbelief), were not discussed in these reviews. Given the complex and heterogenous nature of autism, and the variability in recommendations to support autistic children, it is unsurprising that their child receiving a diagnosis of autism can be stressful to parents (22). In what is often an already stressful period, parents of autistic children report higher levels of parental stress than neurotypical children, and any other disability group (23). Therefore, given benefits of resolution to their child's diagnosis, developing an understanding of factors that may inhibit or support this process may lead to the development of strategies to enable parents to achieve resolution, bringing broad benefits to this population, improving the parent-child relationship, and reducing parental stress.

### 1.1. Current review

The process of resolution allows both parents and children to reach an optimal level of wellbeing (18). The aim of this systematic review was to identify studies from the last 5 years (2017–2022) that identified factors associated with parental resolution of their child's diagnosis of autism. These factors may impact resolution or be impacted by resolution. Resolution was defined as a parent's process of coming to terms with and accepting their child's significant childhood diagnosis (10). To ensure greater depth and understanding of the parental experience, we expanded this definition to include empirical research that specifically investigated “resolution of diagnosis” as well as empirical research that described a process of parental diagnosis separate to this term.

## 2. Methods

Prior to conducting the current review, the International Prospective Register of Systematic Reviews (PROSPERO) was checked for similar reviews, no such reviews were identified. This review was registered with PROSPERO in May 2022 (ID: CRD42022336283). The review adhered to the Preferred Reporting Items for Systematic Reviews and Meta-Analyses (PRISMA) guidelines (24).

### 2.1. Selection criteria and search strategy

A systematic search began in May 2022 utilizing six databases: Scopus, Web of Science, MEDLINE, PsycINFO, and ProQuest. Publication limit of January 2017 onwards to current period (May 2022) was applied. Our search terms (Table 1) reflected definitions of parental resolution or acceptance of child diagnosis, and terms for Autism (Table 1) with combinations of truncated terms searched across all fields (title, abstract, keywords).

**Table 1:** Search terms.

**Search terms included:**
(“Reaction to diagnosis” OR “reaction to the diagnosis” OR “resol^*^ of diagnosis” OR “resol^*^ the diagnosis” OR “resol^*^ of the child^*^ diagnosis” OR “maternal resolution” OR “mother^*^ resolution” OR “acceptance of child^*^ diagnosis” OR “acceptance of the diagnosis” OR “parental resolution” OR “parent^*^ resolution” OR “paternal resolution” OR “father^*^ resolution” OR “(‘rdi' and ‘child' and ‘parent')”) AND (Autis^*^ OR ASD OR Asperger^*^ OR “pervasive development^*^ disorder^*^” OR “spectrum disorder^*^” OR HFASD).

### 2.2. Inclusion and exclusion criteria

To be included in the review, they were (a) published in English, (b) published in peer-reviewed journals or were reviewed dissertations, (c) were empirical studies, and (d) focused on aspects of resolution to diagnosis or acceptance. We required that the population was parents of children with an autism diagnosis, including pre-DSM-5 diagnoses; autism, Asperger's disorder or pervasive developmental disorder not otherwise specified (PDD-NOS), with or without an intellectual disability.

## 3. Results

### 3.1. Literature retrieval and results

**Figure 1:**
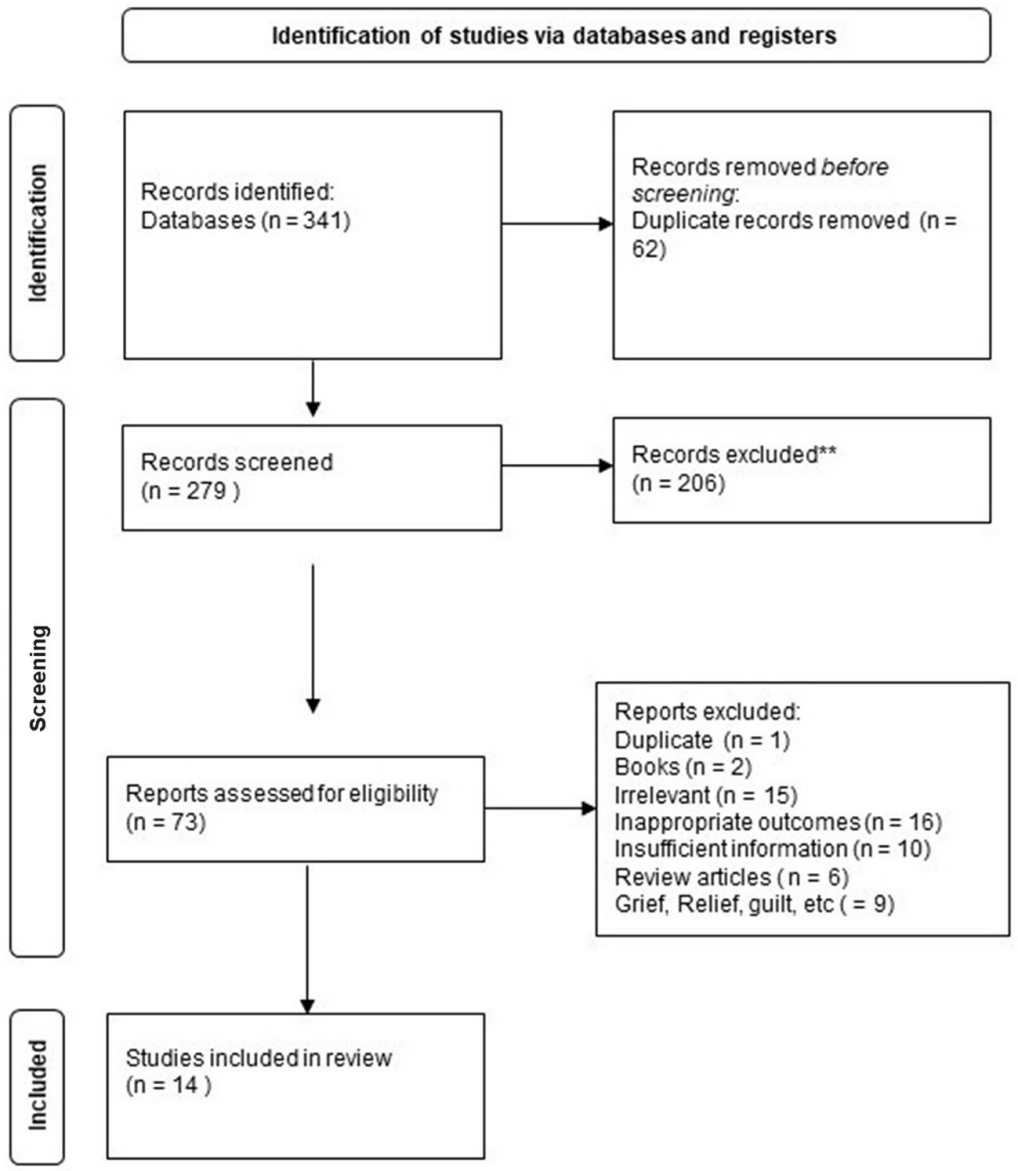
PRISMA flow diagram.

Figure 1 details a comprehensive flow chart of study selection. A total of 341 articles were identified across all six databases. Articles were exported into EndNote X9 (25) for the removal of duplicates, and then imported into Covidence (https://www.covidence.org/) for screening. After title and abstract screening of the remaining 279 papers, 73 studies were included in a full text review. Of these, 59 were excluded for being non-empirical studies, clearly irrelevant, reviews or papers that did not report or measure aspects of resolution or acceptance of a diagnosis or factors associated with acceptance. Three dissertations were identified and included in the review. In total, fourteen papers were relevant and met the research aims and were included in the review.

### 3.2. Sample characteristics

Fourteen studies met the study aims and were included in the final review. The characteristics of these studies are presented in **Table 3**. Studies were conducted in the United States of America (*n* = 8), United Kingdom (*n* = 2), Australia (*n* = 1), Taiwan (*n* = 1), Italy (*n* = 1), and Israel (*n* = 1). A total of 592 participants (*mothers* = 536, *fathers* = 56) with 20 families, in which the study did not mention how many individuals from each family participated in the study. Two Studies focused on Latino participants from America (total *n* = 59), both of these studies compare Latino families with Caucasian families. Of nine studies identifying child gender, 82% of the children diagnosed were males and most studies focused on mothers as participants (*n* = 9), fathers (*n* = 1), both parents (*n* = 3), and families (*n* = 1).

### 3.3. Resolution or acceptance measurement

Measurement varied across studies either measuring resolution, acceptance, or psychological adjustment. A range of different study types were included (*RCT, n* = *1; Longitudinal, n* = *2; Cross-sectional, n* = *1; Mixed methods, n* = *1; Qualitative Studies, n* = *8;* Refer to **Table 3**); 57% of included studies were of a qualitative nature.

For theoretical perspectives, five papers adhered closely to a theoretical definition of resolution, using Marvin and Pianta's (10) model and using the RDI to measure resolution.

Nine studies measured acceptance or a form of acceptance closely resembling resolution. Eight of these studies used semi-structured interviews, with acceptance emerging as a theme amongst experiences of parents, sub-categorizing the process of accepting a diagnosis. One study used the Adjustment Scale (14) to measure acceptance of diagnosis.

Only two qualitative studies clearly indicated resolution (*resolved;* 61.5%). Other studies placed resolution and acceptance on a continuum.

### 3.4. Factors associated with resolution

#### 3.4.1. Child characteristics

De Paz et al. (14) found characteristics of the child, specifically autism severity, had a substantial effect on acceptance or resolution, in which severity was positively associated with acceptance, suggesting that greater condition severity was associated with greater parental acceptance in relation to maternal adjustment to the diagnosis.

#### 3.4.2. Religion, belief, culture

Four studies found that religion, beliefs, and culture impacted acceptance or resolution. All four studies found religion to be a positive coping mechanism positively affecting resolution. Three studies included Arab and Latino participants (see **Table 3**), where culture and associated stigma within cultures was viewed as an inhibitory factor to resolution; across studies, lack of awareness within the community was a common theme associated with belief and culture.

#### 3.4.3. Knowledge and uncertainty

Lack of knowledge and uncertainty was found to be a factor affecting parent resolution in six studies. This factor included specific knowledge about autism symptomology, treatment processes and the overall process of supporting a child with an autism diagnosis. Uncertainty or “the unknown,” was found to stall the process, including uncertainty regarding the future and doubts regarding the final diagnosis (13). Alsayyari (26) found that diagnosis allowed some parents to feel empowered, as they finally understood their child's developmental delays. In Lopez et al. (27) the degree of autism knowledge differed by culture. Although both Latino and Caucasian groups were underinformed, Latino families expressed greater lack of knowledge about autism (27). Finally, parental self-education about autism was found to be a protective factor (13, 16).

#### 3.4.4. Negative emotions

Negative emotions including feelings of grief, denial, shame, and blame emerged as a common theme for 71% of included studies. Negative emotions occurred during initial stages of receiving the diagnosis for most parents but were maintained for parents who had not yet adjusted their internal representations of their child. The more resolved a parent was, the less feelings of guilt, shame, denial, and blame occurred. Ferguson and Vigil (28) found a prolonged period of denial elongated the diagnostic-process in Hispanic families compared to Caucasian families. Further, Reed et al. (29) found mothers who reported a poorer experience of the diagnostic process, tended to be “resolved” in their reaction to the diagnosis. Resolution and acceptance demonstrated lower levels of perceived stress and were associated with lower depressive symptoms (14, 30). Resolved mothers were seen to be emotionally available, reporting less anxiety, depression, better perceptions of their social relationships, and overall increased self-perceived mental and physical health over time, compared to unresolved mothers (4, 31). Contrastingly, Reed and Osborne (31) did not find depression or anxiety to be significantly associated with mothers classified as “unresolved”.

#### 3.4.5. Positive emotions

Positive emotions (e.g., relief) were identified as factors to reaching adjustment faster (14). The adjustment period described was that of redefining goals and expectations of the child and adjusting parent's perceptions of their relationship with their child. Positive experiences and emotional responses were associated with greater acceptance (16). Social support and perceived sense of competence was positively correlated with resolution (30). Furthermore, searching for alternate diagnosis stopped once parents reached a level of acceptance, referring to readjusted expectations of their child's capacity and state of being (17, 32).

#### 3.4.6. Support factor

Heredia-Alvarado and Chen (32) identified positive factors including family support and connection between mother and child, aided the acceptance process. Parents reported a sense of relief upon realizing they were not alone in the community and could access support, decreasing self-blame (17). Hotez (30) found higher levels of resolution and perceived social support were associated with each other. Lopez et al. (27) found support influenced parent involvement in treatment, with Caucasian families tending to feel more supported through services and extended family, while support from children's grandparents was relatively equal. Rabba et al. (16) found increased knowledge surrounding autism was associated with higher support levels. Rafferty et al. (33) found that with the help of family and therapy, a parent was able to gradually progress in their process of accepting their child's diagnosis and the potential effects of this diagnosis on their child's life.

#### 3.4.7. Attunement and insightfulness

Attunement and insightfulness refer to parent's ability to understand the motives behind their child's behavior and emotional experiences in a child-focused and positive manner, resulting in an appropriate response to the child's needs (30, 34). Di Renzo et al. (34) found that more accepting parents were more attuned to their child during play interaction and were better able to see things from the child's perspectives, in comparison to non-resolved parents, with 30% of resolved/insightful parents being attuned to their child's needs. Furthermore, a Randomized Control Trial (RCT) by Hotez (30) testing a playtime intervention with parents and children which examined attachment, parental perception, and parent insightfulness, found that the highest proportion of mothers (38.2%) were unresolved and non-insightful. However, this RCT was the only one identified in our search that met the inclusion criteria.

For further categorization of studies and descriptive data, please see Table 2.

**Table 2:** Factors associated with resolution.

**Factors associated with resolution**	**References**
Child characteristics	Severity of diagnosis (14 )
Religion, belief, culture	(26–28, 32)
Knowledge and uncertainty	(13, 16, 26, 32, 33)
Negative emotions	(13, 14, 17, 26–33)
Positive emotions	(4, 14, 16, 31, 32)
Support	(16, 17, 27, 30, 32, 33)
Attunement and insightfulness	(30, 34)

## 4. Discussion

Previous research suggests that resolution and acceptance of a child's diagnosis has positive effects on parent-child relationships and wellbeing (6). Given the increased stress and mental health challenges often reported by parents of autistic children (i.e., compared to parents of children with other significant childhood diagnoses), the present systematic review examined parental resolution of their child's diagnosis of autism, specifically factors that may support or present barriers to resolution, and potential benefits brought by resolution to diagnosis.

Six factors emerged across the fourteen studies, five of which impacted resolution and one which was determined to be an outcome of resolution. Factors included child characteristics (severity of autism), religion, belief and culture, knowledge and uncertainty, negative emotions (including denial, shame, guilt), positive emotions and factors (such as family support and emotional availability), and attunement and insightfulness.

An interesting finding was that severity of diagnosis was associated with greater resolution. As severity of diagnosis likely comes with greater challenges, this finding seems somewhat counterintuitive. In the broader literature, the impact of severity of diagnoses on resolution is mixed, with findings supporting a negative association or no association at all (6). For parents of autistic children in one study (14), severity seemed to aid in the resolution process suggesting that severity may be associated with greater acceptance because there is less opportunity for hope that the diagnosis is false, or denial of the condition, thereby facilitating the acceptance process. Although it is important to note that only one study (14) found severity as an associated child characteristic of acceptance in the current review, this finding is consistent with Sher-Censor et al. (6) who found less severe symptoms were associated with resolution.

The role of negative and positive emotions has consistently been shown to be associated with resolution in other childhood diagnoses (6). An interesting finding from our study was the relationship between culture and beliefs and emotions. Negative emotions were found to be associated with culture and ethnic background such that Latina and Hispanic mothers were more likely to experience an increased sense of guilt once receiving the diagnosis, whereas Caucasian families reported a sense of relief and were more likely to have a briefer adjustment period (27, 32). This complex interplay between emotion and culture may not only impact the initial reaction to the diagnosis, but also length of diagnostic resolution process and ultimately parental resolution. Thus, understanding culture and its potential impact on resolution is important for professionals supporting parents during the diagnostic process.

Uncertainty and lack of knowledge of autism was associated with negative emotions and was a common theme inhibiting resolution and acceptance. This association may be explained by Chao et al. (17) who coined the phrase “anxious searching”, whereby parents sought a second opinion when they did not accept the initial diagnosis. Alternatively, Ferguson and Vigil (28) suggested factors including parents' negative attitudes toward autistic people, and lack of awareness about autism by family and friends, might lead to apprehensiveness, worry, embarrassment, and being generally upset by their child's diagnosis.

These adverse feelings are not surprising, given that broader disability literature indicates that the diagnostic process represents a challenging and confusing period for parents. With regards to autism, parents have reported that medical professionals often vary in their knowledge and experience with autism (35, 36), or predominately focus on negative factors or limitations associated with the diagnosis (22). Framing the diagnosis as overly negative or not discussing strengths or solutions, has been shown to negatively impact the parents' experience of the diagnostic process as well as their understanding of autism (37, 38). As resolution requires developing a realistic appraisal of the child's prognisis and potential challenges, ensuring that parents have information surrounding the diagnosis is an important step toward resolution.

Another interesting finding was the interplay between time and the experience of negative emotions. Across most studies, negative emotions were more prominent amongst parents when they initially received their child's diagnosis. This finding is consistent with research involving diagnoses of other disabilities (33) and highlights that resolution is a process. Moreover, while negative emotions may be present at the beginning of the process, with greater support and knowledge about autism, including adopting a strength-based approach, these negative emotions can decrease (28), thereby facilitating acceptance of the child's diagnosis.

Despite the studies in this review similarly describing the process of acceptance, across the studies, they were often labeled differently. Thus, there were inconsistencies across the studies in terms of the interpretation of the process of resolution. For example, Da Paz et al. (14) understood acceptance to be dichotomous, creating different stages of acceptance, while Lazarus et al. (12) understood it to be a more sensitive and complex continuous scale. Similarly, Chao et al. (17) defined “acceptance” as referring to the timepoint of receiving the diagnosis, while the “process of resolution” was described as an adjustment period whereby parents adjust their goals and expectations for their child. Comparatively Shih (13) included Expectation Management Theory of Acceptance whereby expectational adjustment, the process prior to acceptance, paralleled the construct of resolution. Thus, the present review highlights the role different theoretical positions or perspectives have on understanding the construct of resolution.

### 4.1. Quality of studies

The present review identified only one randomized control trial (RCT), while 57% of studies were of a qualitative nature. Although, qualitative studies can lead to greater insight of the topic matter (26), qualitative studies are required to explore relationships between factors and determine causal mechanisms. Furthermore, relationships between factors reported as significant were often of small effect size (9, 14), with few reporting larger effect sizes between factors (30) (please see Table 3 for a summary of the strengths and weaknesses of each study included in the review).

**Table 3:** Summary of reviewed studies.

**References**	**Design**	**Country**	***N* (parents)**	**Aim**	**Measurement**	**Factors associated with resolution**	**+**	**–**	**Effect size**
Alsayyari (26)	Qualitative (dissertation)	USA	5 Arab American mothers	Overall experience of Arab Americans parents with children diagnosed with autism	Acceptance: Developed a semi-structured interview (two languages)	Education, religion; beliefs and culture	Discussed factors affecting acceptance of diagnosis and characterizes acceptance	Resolution was not specifically identified	Not reported or not applicable
Chao et al. (17)	Qualitative	TAIWAN	15; 14 mothers and 1 father	Understand the parents subjective experience, emotions, and adjustments encountered before, during, and after diagnosis after the process of obtaining a confirmed diagnosis of ASD for their child	Acceptance: Developed a semi-structured interview	Accepting their role as a parent and autism knowledge allowed parents to feel empowered	Phenomenological approach providing a detailed and comprehensive understanding of complex parent emotions	Parents access to resources and support systems in place were not controlled for, reducing generalizability; did not characterize the diagnostic process	Not reported or not applicable
Da Paz et al. (14)	Longitudinal	USA	90 mothers	Maternal adjustment regarding acceptance and despair	Acceptance: Novel 30-item self-report questionnaire: adjustment to the Diagnosis of Autism (ADA)	Acceptance associated with lower depressive systems across time and cross-sectionally	Explored the relationship between acceptance and despair	Does not identify resolution specifically; describes the process of resolution; used a novel 4-point Likert scale	Correlation: Acceptance with autism severity (*r* = 0.25; *p* = 0.02) Acceptance with lower depressive symptoms (*r* = – 0.34; *p* < 0.001) Regression: Acceptance over time with reductions in depressive symptomology (β = – 0.30; *p* = 0.05)
Di Renzo et al. (34)	Cross sectional	ITALY	50; 26 mothers and 24 fathers	Parent child attunement during play, parental insightfulness, and acceptance of diagnosis	Resolution: RDI	More accepting of autism diagnosis associated with more attuned during play interaction with child and ability to see things from the child's perspective; education associated with resolved parents	Measured multiple variables and association between resolution and insightfulness; multiple measures (recording, interview)	Small sample size; use of non-validated measure; observational and *via* interview may affect generalizability	Association with educational level and resolved/insightful statistically significant (LR = 10.269, *df* = 4, *p* < 0.05)
Ferguson and Vigil (28)	Qualitative	USA	20 families; 10 Hispanic families; 10 non-Hispanic white families	Compare Hispanic and non-Hispanic white families both from low SES experiences regarding their autistic child	Acceptance: Developed a semi-structured interview (adapted)	Prolonged period of denial in Hispanic families affecting seeking diagnosis in comparison to Caucasian families who reached acceptance quicker; knowledge; religion	Compared SES between two ethnic backgrounds; detailed data collection to evaluate demographics and differentiating factors	Acceptance was a secondary focus and resolution was not explicitly mentioned; demographic information on each participant was not included	Not reported or not applicable
Heredia-Alvarado and Chen (32)	Qualitative (dissertation)	USA	15 first-generation Latino mothers	Experience of first-generation mothers with children diagnosed with autism	Acceptance: Developed a semi-structured interview	Knowledge; negative factors: painful, denial, self-blame/guilt, shame, depression/isolation; protective factors: family support, connection between mother and child	Included comprehensive sub-themes of factors affecting acceptance of diagnosis and description of accepting diagnosis	Did not characterize resolution, only identified acceptance	Not reported or not applicable
Hotez (30)	RCT (dissertation)	USA	70 mothers: 25 (36.2%) resolved; 44 (63.8%) unresolved	Identify baseline associations between insightfulness and resolution with parental cognitions and emotions, child characteristics and family demographic	Resolution: RDI	Treatment; stress and perceived sense of competence and understanding of child development	RCT presented excellent internal validity	Employed novel method of conceptualizing insightfulness and resolution as continuous variables	Not reported or not applicable
Lopez et al. (27)	Qualitative	USA	96; 44 Latino mothers and 52 White mothers	Explore how Latino parents react to their children's diagnosis compared to Caucasian parents and how family and culture play a role in their resolution process	Acceptance: Developed a semi-structured interview	Knowledge; guilt; culture	Large sample size; associated common emotions such as guilt and relief	Semi-structured interview used to measure resolution; does not identify how many parents were resolved/unresolved	Not reported or not applicable
Rabba et al. (16)	Qualitative	AU	13; 10 mothers and 3 fathers	Parental experiences and needs following child's early diagnosis	Acceptance: Developed a semi-structured interview	Sharing the diagnosis; emotional response	Extended research on autism resolution within Australia; themes had sub-themes exploring nuances of emotional response such as uncertainty	Resolution was a secondary focus *via* a theme identified; interview questions were developed no information on validity; different methods of data collection; four participants attended face-to-face focus group and nine in semi-structured phone interviews	Not reported or not applicable
Rafferty et al. (33)	Qualitative	USA	28 fathers; autism (*n* = 12) and autism/ID (*n* = 16)	Examine father-child relationships and perceptions of parenting roles	Acceptance: Developed a semi-structured interview	Uncertainty of the “unknown”; denial emotional struggles; relief with better understanding of child's behavior; acceptance and love for the child as they are	Focus on fathers; good range of ethnic backgrounds	Does not explore resolution in detail, only identifying it within a theme, explores acceptance	Not reported or not applicable
Reed and Osborne (31)	Longitudinal	UK	84 mothers: 52 (62%) “resolved”; 32 (28%) “unresolved”	Whether reactions to diagnosis are associated with health status for mothers at the time of receiving the diagnosis and whether diagnostic resolution is associated with health status changes over time	Resolution: RDI	Resolved mothers had less anxiety, depression; better perceptions of their social relationships and of their physical health; less problems with immune function; increase in self-perceived health	Children assessed by a multidisciplinary team	Results obtained from a single clinic; lack of prior information on physical and mental health or prior knowledge	Significant effect of resolution on self-perceived physical health, somatic health and immune functioning (ηp^2^ = 0.278; ηp^2^ = 0.343; ηp^2^ = 0.156)
Reed et al. (29)	Longitudinal	UK	67 mothers; 41 (61%) resolved and 26 (39%) unresolved	Assess the diagnostic process associated with reaction to a child's diagnosis	Resolution: RDI	Age of child at start of diagnostic process and perceived speed; better interpersonal skills	A range of ethnic and demographic backgrounds	Sample drawn from one site; research or knowledge mothers had prior to receiving diagnosis was not considered	Stepwise logistic regression: mothers' perceptions was significant, −2LL = 57.81, χ2_(5)_ = 23.26, *p* < 0.001
Sher-Censor et al. (4)	Mixed Methods	ISRAEL	46 mothers Arab-Israeli families	Interplay of mother's coherent representations of their child, resolution of autism diagnosis and emotional availability to child	Resolution: RDI	Resolved mothers are emotionally available	Time for adjustment was allowed (6 months); mixed methods design allowing for detailed and generalizable data	Possible effects of maternal representation on maternal behavior when interacting with their child	Not reported or not applicable
Shih (13)	Qualitative (dissertation)	USA	13 mothers	Process of acceptance in mothers	Acceptance: Developed a semi-structured interview	Prior recognition of child limitations; uncertainty; methods of adjustment	Discusses in detail different types of acceptance	Did not characterize resolution, instead acceptance, with resolution acknowledged in association to expectation adjustment theory and acceptance; locally recruited participants; relied heavily on interview data	Not reported or not applicable

### 4.2. Limitations and future directions

Some papers referred to resolution as an adjustment period (14). Nonetheless, discrepancies were apparent in framing of key factors and theoretical positions. More research and theoretical development are needed to ensure research is guided by a coherent and cohesive definition of resolution and acceptance. Our review did not explicitly include papers regarding grief, shame, loss and even relief, which often occur at later stages of the acceptance process. Future research that explores potential relationships between these factors and resolution are needed. Additionally, few studies examined multifaceted, interactive aspects of resolution. For example, the acceptance of one's role as a parent of an autistic child is a necessary part of accepting a child's care needs. Furthermore, factors such as knowledge and educational levels are also likely to interact to influence resolution. One important step for future research will be the development of valid and reliable measures of resolution, that are grounded in theory. Finally, future research examining diagnostic resolution and other constructs (e.g., hope), or how resolution may affect the disclosure of autism to others or to the child themself, or long-term effects on the parent-child relationship, are needed.

## 5. Conclusion

Receiving a child's autism diagnosis can be a stressful time (3) and resolution contributes to the wellbeing and the development of healthy parent-child relationships (6). This review identified articles examining factors contributing to diagnostic resolution associated with a child's diagnosis of autism. Although the identified studies supported associations between resolution and family wellbeing, the overall quality of studies was poor, relying on qualitative or correlational designs. Future research that better synthesize different approaches and tests theoretical processes with large, well-designed longitudinal studies, is therefore required.

## Data availability statement

The original contributions presented in the study are included in the article/supplementary material, further inquiries can be directed to the corresponding authors.

## Author contributions

VN, SB, and DH conceived of the study. VN conducted the literature review, completed the systematic search, and analyzed the data under the supervision of SB and DH. VN wrote the first draft. All authors discussed the result and contributed to the writing of the final manuscript.
